# Undernutrition in young children with congenital heart disease undergoing cardiac surgery in a low-income environment

**DOI:** 10.1186/s12887-023-04508-x

**Published:** 2024-01-23

**Authors:** Smith Robyn, Ntsiea Veronica, Brown Stephen, Potterton Joanne

**Affiliations:** 1 Department of Physiotherapy, Faculty of Health Sciences, University of the Witwatersrand, Johannesburg, South Africa; 2 Department of Pediatrics and Child Health, University of the Free State, Bloemfontein, South Africa; 3 School of Health and Rehabilitation Sciences, University of the Free State, Bloemfontein, South Africa

**Keywords:** Congenital heart disease, Cardiac surgery, Malnutrition, Undernutrition, Failure to thrive, Feeding and swallowing difficulties, Low-income environment, Central South Africa

## Abstract

**Background:**

Malnutrition (undernutrition) in children with congenital disease (CHD) is a notable concern, with preoperative and persistent growth failure post-cardiac surgery contributing to poorer outcomes. Poor growth in children with CHD in low-income environments is exacerbated by feeding difficulties, poverty, delayed diagnosis, and late corrective surgery. This study describes and compares the growth of young children with CHD undergoing cardiac surgery in central South Africa from before to 6-months after cardiac surgery.

**Methods:**

Children 30 months and younger, with their mothers, were included in this prospective observational descriptive study. Weight- height-, and head circumference-for-age z-scores were used to identify children who were underweight, stunted and microcephalic. Z-scores for growth indices were compared from baseline to 3-months and 6-months post-cardiac surgery. Changes in growth over time were calculated using a 95% confidence interval on the difference between means. Linear regression was used to determine the association between growth and development, health-related quality of life and parenting stress respectively.

**Results:**

Forty mother-child pairs were included at baseline. Most children (*n* = 30) had moderate disease severity, with eight children having cyanotic defects. A quarter of the children had Down syndrome (DS). Twenty-eight children underwent corrective cardiac surgery at a median age of 7.4 months. Most children (*n* = 27) were underweight before cardiac surgery [mean z-score − 2.5 (±1.5)], and many (*n* = 18) were stunted [mean z-score − 2.2 (±2.5)]. A quarter (*n* = 10) of the children had feeding difficulties. By 6-months post-cardiac surgery there were significant improvements in weight (*p* = 0.04) and head circumference (*p* = 0.02), but complete catch-up growth had not yet occurred. Malnutrition (undernutrition) was strongly associated (p = 0.04) with poorer motor development [Mean Bayley-III motor score 79.5 (±17.5)] before cardiac surgery. Growth in children with cyanotic and acyanotic defects, and those with and without DS were comparable.

**Conclusion:**

Malnutrition (undernutrition) is common in children with CHD in central South Africa, a low-income environment, both before and after cardiac surgery, and is associated with poor motor development before cardiac surgery. A diagnosis of CHD warrants regular growth monitoring and assessment of feeding ability. Early referral for nutritional support and speech therapy will improve growth outcomes.

## Background

Congenital heart disease (CHD) is the most common congenital abnormality affecting nearly one in every hundred children born [1]. Approximately one in three children born with CHD will require surgical or catheter-based intervention early in life [2]. Earlier diagnosis and considerable advances in medical and surgical management see most children born with CHD today survive [3]. Impaired growth has become a notable concern for children with CHD both before and after cardiac surgery, irrespective of the type of cardiac defect and the presence or absence of cyanosis [4–6]. Most children with CHD born at term gestational age have normal anthropometric indices, however soon after birth many begin to exhibit growth challenges including deficits in weight, height and head circumference that place them at high risk for malnutrition and failure to thrive [7–9]. The terms malnutrition and undernutrition are often used interchangeably. Malnutrition encompasses three broad groups of conditions including undernutrition, overnutrition and micronutrient-related malnutrition. Undernutrition refers to the insufficient intake of calories and nutrients and includes underweight (low weight-for-age), stunting (low height-for-age) and wasting (low weight-for-height) [10].

It is recognized that the cause of malnutrition, specifically undernutrition, in children with CHD is multifactorial. Causes include the underlying cardiac abnormality itself, hemodynamic factors, chronic hypoxemia, inadequate calorie intake, increased energy expenditure in relation to intake (hypermetabolism), malabsorption, co-occurring genetic comorbidity and feeding and swallowing difficulties (FSDs) [11–14]. Difficulties in coordinating breathing with swallowing due to shortness of breath resulting from congestive cardiac failure (CCF) or respiratory infection, neurobehavioral challenges, fatigue due to poor physical endurance, dysphagia, gastroesophageal reflux disease (GERD), cleft lip palate (CLP), oral aversion, delayed acquisition of feeding skills and vocal cord dysfunction caused by laryngeal nerve injury during cardiac surgery contribute to FSDs [7, 15, 16]. FSDs result in inadequate calorie intake, making the early recognition and management thereof important to improve growth outcomes [15, 16]. Malnutrition prior to cardiac surgery contributes to delayed cardiac surgery, and poorer short and long term outcomes including longer hospital length of stay (HLOS), higher risk of infection, poorer clinical and neurodevelopmental outcomes, and poorer health-related quality of life (HRQOL) [4, 5, 17–20]. Furthermore, growth failure and FSDs contribute to increased levels of parenting stress [19, 21–25]. Similar to other children, children with CHD are also at risk for malnutrition secondary to social and economic factors [26, 27].

The reported overall prevalence of moderate malnutrition in young children with CHD in high income countries (HICs) ranges from 21 to 29% [4, 6, 13, 17]. A recent systematic review and meta-analysis on the prevalence on malnutrition in children with CHD found that 27.4% of all children with CHD were underweight and 24.4% were stunted [17]. This is significantly higher than the estimated prevalence of underweight of 6.1% and stunting of 7.6% reported for the global population of otherwise healthy same-aged children [28, 29]. Admittedly early intervention for CHD is shown to improve catch-up growth [11, 30].

Published data on malnutrition in children with CHD living in low-to-middle income countries (LMICs), though far scanter, suggests the prevalence of growth failure is substantially higher and the extent of the malnutrition greater than for children living in HICs [17]. Moderate malnutrition is said to occur in more than half of children with CHD living in LMICs, and severe malnutrition is noted to be as high as 60% in some cases [14, 30–33]. Overall rates of malnutrition as high as 90% have been reported in some studies [14, 31–39]. It is apparent that poverty, delayed diagnosis and later age at corrective surgery exacerbate the extent of the growth failure in children with CHD in LMICs [11, 40].

More than a third of the 1.5 million children born annually with CHD live on the African content, with around 70% of these children requiring cardiac intervention to survive or improve their HRQOL [41, 42]. Cardiac surgery and interventional cardiology capacity is severely constrained on the African content, and in South Africa, resulting in overburdened cardiac services and extended waiting periods for cardiac intervention which increases the risk of undernutrition [14, 41, 43–49].

A small number of African studies over the last decade have investigated the growth outcomes of children with CHD, including in Nigeria [14] Uganda [32, 34], Ethiopia [33, 35] and Egypt [36, 37]. No published data is however available on the growth outcome of young children with CHD in South Africa (SA), despite SA having the most established pediatric cardiac program on the continent [47, 50, 51].

The lack of information on growth outcomes for children with CHD in SA is a concern. Despite being an upper middle income country more than six out of ten South African children are known to be multidimensionally poor, with two thirds of children under the age of 5 years living below the food poverty line, in households where there is insufficient money available to meet even basic nutritional needs [52–55]. Moreover, a third of young children living in central SA (Free State and Northern Cape provinces of SA, and the neighboring country of Lesotho) are said to be chronically malnourished [56, 57]. Based on available evidence it can be postulated that being born with CHD in a low-income environment such as central SA is likely to increase the likelihood and severity of malnutrition [11, 40, 53]. The nature and extent of malnutrition in children with CHD in SA needs to be established in order to plan appropriate nutrition interventions and feeding therapies to support optimal nutrition and enhance post-operative outcomes [9].

Better understanding the nature and extent of malnutrition in children with CHD living in low-income environments is not only of importance to LMICs. Surprising to many, child poverty in HICs such as the United States (US) and United Kingdom (UK) is high and continues to rise. Over 12.5 million children in the US and 2.6 million children in the UK live in food insecure households [58, 59].

This study aimed to describe and compare the growth outcomes of young children with CHD undergoing cardiac surgery in central SA from before cardiac surgery to 3-months and 6-months after cardiac surgery.

## Methods

Forty consecutive children with CHD, 30 months and younger, and their mothers were recruited into this prospective observational descriptive study at a tertiary level hospital (specialist center) in central SA over a 17-month period. Neonates, children who were critically ill and those who had undergone previous, or emergency cardiac surgery were excluded. Children with genetic disorders, which in our study were all Down syndrome (DS), were included in the study sample as a group of special interest, as information on the impact of a genetic disorder with CHD on growth outcomes is lacking [60]. This article reports on the growth outcomes of children with CHD as part of a larger single center longitudinal study [61].

Ethical clearance was obtained from the Health Sciences Research Ethics Committee of the University of the Free State (ECUFS 177/2013) and the Committee for Research on Human Subjects at the University of Witwatersrand (M131056). Mothers provided informed consent for their own and their child’s participation in the study, and all participant information was kept confidential. The study was conducted in line with the ethical principles outlined in the Declaration of Helsinki [62].

Sociodemographic information including maternal age, parent education level, occupation, and number of siblings in the family was collected using a verbally administered questionnaire. Medical and surgical information was collected from the child’s medical record. Routine clinical examination was conducted by the pediatric cardiologist and cardiothoracic surgeon. A clinical diagnosis of a genetic disorder was made by the consulting pediatrician and confirmed by genetic testing. For this study the child’s CHD was considered complicated when it was associated with reversible pulmonary hypertension (PHT) and/or congestive cardiac failure (CCF). Severity of the cardiac disease was rated using the Cardiologists Perception of Medical Severity Scale [63], and socioeconomic status calculated using the Hollingshead Index of Social Position [64].

Weight, height, and head circumference were measured using standard protocols, and z-scores determined using the World Health Organization (WHO) child growth standards and DS-specific growth charts. Children with z-scores below − 2 were considered undernourished (i.e., those who were underweight and stunted) and microcephalic [65–67].

Developmental status was assessed using the Bayley Scales of Infant and Toddler Development, third edition (Bayley-III) [68]. Parenting stress was determined using the Parenting Stress Index Short-Form [69], and HRQOL using the Pediatric Quality of life Inventory [70]. All key measures were repeated at baseline (prior to cardiac surgery) and at 3-months and 6-months post-cardiac surgery.

On average 180 children are treated at the cardiac center annually, with 60 children 30 months and younger undergoing cardiac surgery. The sample size achieved of 40 mother-child pairs had a 90% power to detect a difference in means of 10 based on a standard deviation (SD) of 15, considering a possible 15% loss to follow-up and non-compliance of 10% with the Bayley-III as the main outcome measure.

Sample characteristics and clinical variables are presented as means with SD and medians with ranges for continuous data and frequencies with percentages for categorical data. To determine if catch-up growth occurred after cardiac surgery, z-scores were compared for all growth indices from baseline to 3-months and from baseline to 6-months post-cardiac surgery. Changes in outcomes over time were calculated using a 95% confidence interval on the difference between means.

A linear regression analysis was conducted to determine if variables which included growth status (weight-for-age), and where indicated feeding difficulties, were predictive of key outcomes including neurodevelopment (gross motor, fine motor and cognitive), HRQOL and parenting stress using two-way analysis of variance (ANOVAs). The effects used were the subcategories under each outcome. Interaction effects were also tested where appropriate. Testing was done at a 0.5 level of significance.

The primary aim was to report on growth outcomes for the entire sample; however, growth outcomes of children with cyanotic heart defects, and those children with genetic comorbidity (DS in all cases in our study) were of interest. The difference between growth outcomes for children with cyanotic and acyanotic defects and those with and without DS were calculated using a two-tailed t-test.

Furthermore, the difference in medical profile and cardiac surgery outcomes (intensive care and HLOS, cardiopulmonary bypass time and postoperative complications) for children with and without undernutrition were calculated on the difference in means using a two-tailed t-test and the difference in proportions using the Chi-squared test. The assumption was that the data followed a normal distribution as the sample size was too small for a proper test of normality.

## Results

**Fig. 1:**
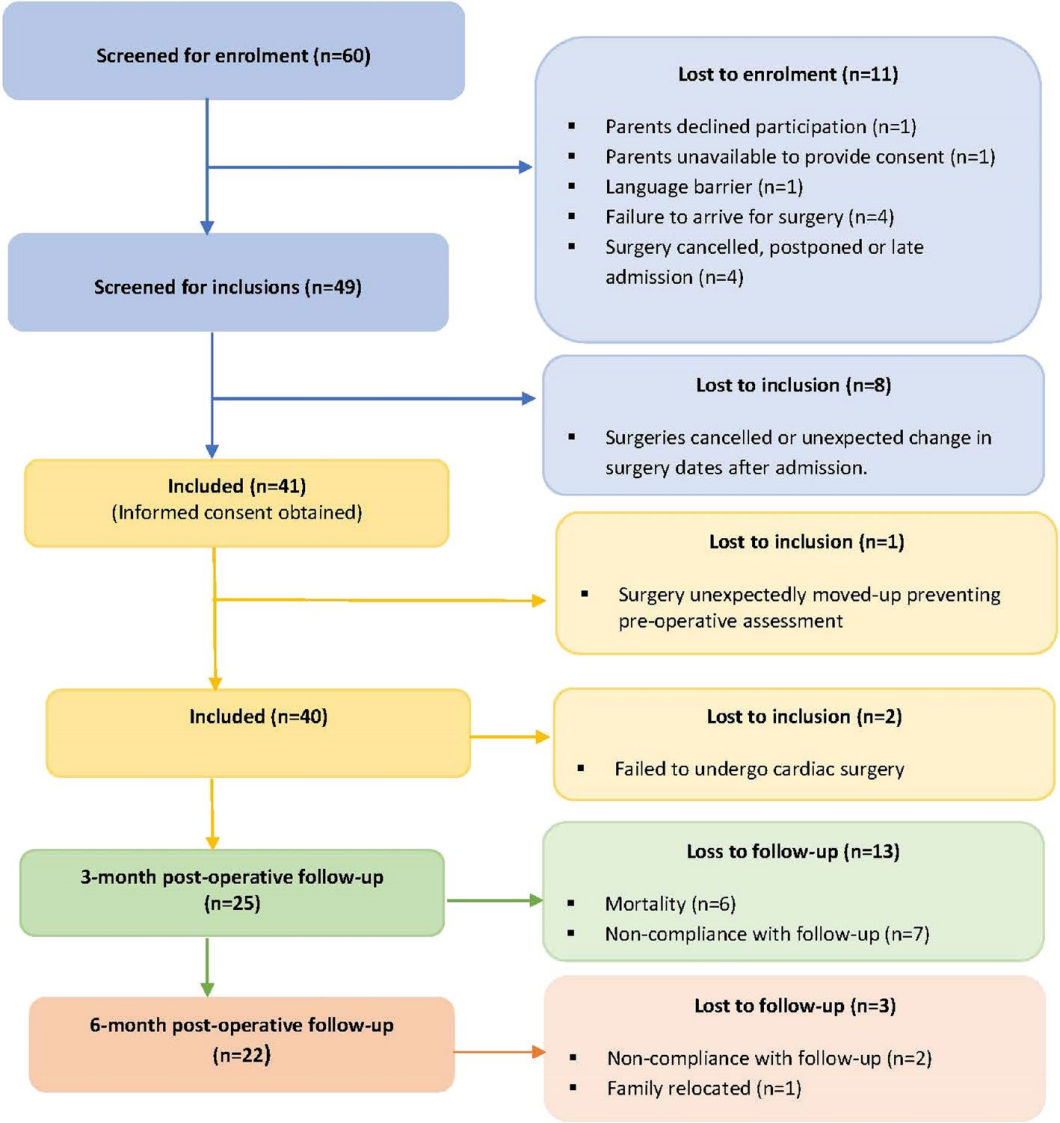
Participant recruitment and study attrition

Baseline data was collected for 40 mother-child pairs. Two mother-child pairs were excluded after baseline as the children failed to undergo cardiac surgery. In addition, loss to follow-up resulted in data only being collected for 25 mother-child pairs at 3-months and 22 mother-child pairs at 6-months post-cardiac surgery (Fig. 1).

The mean age of mothers was 29.6 (±8.0) years. In most instances the mother fulfilled the role of the primary caregiver (*n* = 39), and on average the children in our study had one sibling. Most families (*n* = 35) were from low-income environments. Mothers tended not to work outside of the home (*n* = 33) and took on most of the domestic and childcare responsibilities. Nearly a third of the fathers in our study were unemployed (Table 1).

**Table 1:** Child and family variables

Child related variables	Findings	Parent related variables	Findings
Nature cardiac defect		**Age of mother (years)**	
*Acyanotic*	**32 (80%)**	Median [Range]	30 [16 – 43]
• VSD	15 (37.5%)	Mean (SD)	29.6 (±8.0)
• AVSD	9 (22.5%)		
• PDA	4 (10%)		
• COA	3 (7.5%)		
• AS	1 (2.5%)		
*Cyanotic*	**8 (20%)**		
• DORV	3 (7.5%)		
• TGA	1 (2.5%)		
• TAPVD	1 (2.5%)		
• HLHS	1 (2.5%)		
• TOF	1 (2.5%)		
• TA	1 (2.5%)		
Complicated CHD		**Primary caregiver**	
Uncomplicated	12 (30%)	Mother	39 (97.5%)
Complicated PHT and/ or CCF:	28 (70%)	Father	1 (2.5%)
• PHT	6 (21.4%)		
• CCF	17 (60.7%)		
• PHT and CCF	5 (17.9%)		
Presence genetic abnormality		**Number of siblings**	
CHD alone	30 (75%)	Median [Range]	1 [0 – 4]
CHD with Down syndrome	10 (25%)	Mean (SD)	1.2 (±1.1)
Age baseline assessment (months)		**Mean level of education**	
Median [Range]	7.4 [1.4 – 20.9]	Mothers	Grade 9-11
Mean (SD)	9.2 (± 5.4)	Fathers	Grade 9-11
Gestational age (weeks)		**Unemployed**	
Median [Range]	38 [31 – 41]	Mothers	33 (85%)
Mean (SD)	37.5 (±2.0)	Fathers	13 (32.5%)
Birth weight (grams)		**Socioeconomic status**	
Median [Range]	2800.0 [1640 – 3950]	Mean socioeconomic status	Lower income
Mean (SD)	2878.5 (±608.3)	Lower income	35 (87.5%)
Age at first cardiac surgery (months)		**Distance services (kilometers)**	
Median [Range]	7.5 [1.4 – 20.9]	Median [Range]	162.4 [12 – 782.8]
Mean (SD)	9.4 (±5.5)	Mean (SD)	202.8 (±188.7)
HLOS post-surgery (days)		**Feeding difficulties present**	10 (25%)
Median [Range]	9 [3 – 108]		
Mean (SD)	14.7 (±18.8)		

*VSD* ventricular septal defect, *AVSD* atrioventricular septal defect, *PDA* patent ductus arteriosus, *DORV* double outlet right ventricle, *TGA* transposition of the great arteries, *TAPVD* total anomalous pulmonary venous drainage, *HLHS* hyperplastic left heart syndrome, *TA* tricuspid atresia, *TOF* Tetralogy of Fallot, *AS* aortic stenosis, *CCF* congestive cardiac failure, *CHD* congenital heart disease, *HLOS* hospital length of stay, *PHT* pulmonary hypertension, *SD* standard deviation

At baseline, most children (*n* = 30) had moderate cardiac disease severity, with 20% having cyanotic defects (*n* = 8). Of the 28 children with complicated CHD, 6 children (21.4%) presented with reversible PHT, 17 children (60.7%) with (CCF), and there were 5 children (17.9%) with PHT and CCF. A quarter of the children (*n* = 10) were diagnosed with DS. Most of the children (*n* = 26) underwent open-heart surgery in infancy, with definitive correction being done in most cases (*n* = 28). The median age at first cardiac surgery was 7.4 months (with a range of 1.4 - 20.9 months) (Table 1).

Twenty-seven (68%) children were underweight prior to cardiac surgery. Of these children 10(25%) were moderately underweight and 17(42.5%) were severely underweight. Five children (12.5%) were moderately stunted, and 10 children (32.5%) were severely stunted. A quarter of the children (*n* = 10) had microcephaly (Table 2). Furthermore, a quarter of mothers (*n* = 10) reported that their children had feeding difficulties prior to cardiac surgery. Reported feeding difficulties included struggling to suck, swallow and breathe during feeds due to shortness of breath, easy fatiguability during feeding, and GERD. Several studies have reported an association between CHD and CLP [71–73]. A previous study reported that 14.5% of children treated for CLP at the study site had a known heart defect [71]; however, none the children in the current study had a CLP. Additionally, eighteen children (45%), including all ten children with DS, presented with hypotonia prior to cardiac surgery (Table 1).

**Table 2:** Medical profile and cardiac surgery outcomes for children with and without undernutrition

Descriptor	Undernourished	Adequately nourished	Difference in groups
CARDIOLOGY OUTCOMES (*N* = 40)			
Sample size	**(*n*** **= 27)**	**(*n*** **= 13)**	
Type of defect			
• Cyanotic	8 (29.6%)	2 (15.4%)	*P* = 0.33
• Acyanotic	19 (70.4%)	11(84.6%)	
Primary cardiac diagnosis			
• PDA	4	0	
• VSD	10	6	
• AVSD	5	3	
• COA	2	1	
• TOF	1	0	
• HLHS	1	0	
• TGA	1	0	
• TA	1	0	
• DORV	2	1	
• TAPVD	0	1	
• AS	0	1	
Presence of genetic disorder (DS)	6 (22.2%)	4 (30. 8%)	*p* = 0.56
*Complicated CHD*	21 (77.8%)	9 (69.2%)	*p* = 0.56
• Reversible PHT	13	4	
• CCF	6	3	
• CCF and reversible PHT	2	2	
*Disease severity*	Mean severity: moderate	Mean severity: moderate	
mild	1 (3.6%)	3 (23.1%)	
moderate (correctable surgery)	18 (66.6%)	9 (69.2%)	
moderate to severe (more surgeries)	7 (25.9%)	1 (7.7%)	
severe (uncorrectable or palliated)	1 (3.7%)	0	
FSDs	7 (25.9%)	3 (23.1%)	*p* = 0.85
FSDs with CCF	6 (85.7%)	1 (33.3%)	*p* = 0.28
SURGICAL OUTCOMES (*N* = 38)			
Sample size	**(*n*** **= 25)**	**(n = 13)**	
*Age at surgery (months)*			
Mean and SD	11.4 (7.4)	9.3 (6.9)	*p* = 0.40
Median and Range	11.5 [1.9 -31.5]	6.8 [1.1- 23.9]	
*ICU length of stay (days)*			
Prolonged stay (≥7 days)	10 (40%)	4 (30.7%)	*p* = 0.58
Mean (SD)	8.7 (6.6)	5.8 (2.2)	*p* = 0.13
Median and Range	6 [3-28]	5.5 [3-10]	
*Hospital length of stay (days)*			
Prolonged (≥ 14 days)	9 (36%)	7 (53.8%)	*p* = 0.30
Mean and SD	22.5 (24.9)	15.7 (11.6)	*p* = 0.36
Median and Range	11 [6 -108]	12.5 [4-49]	
*Cardiopulmonary bypass*	15 (60%)	11 (84.6%)	*p* = 0.14
*Cardiopulmonary bypass time (min*)			
Mean and SD	119.1 (63.1)	100 (47.8)	*p* = 0.41
Median and Range	105 [55-300]	92.5 [41-190]	
*Postoperative complications*	8 (32%)	2 (15%)	*p =* 0.26

*PDA* patent ductus arteriosus, *VSD* ventricular septal defect, *AVSD* atrioventricular septal defect, *COA* coarctation of the aorta, *TOF* Tetralogy of Fallot, *HLHS* hypoplastic left heart syndrome, *TGA* transposition of the great arteries, *TA* tricuspid atresia, *DORV* double outlet right ventricle, *TAPVD* total anomalous pulmonary venous drainage, *AS* aortic stenosis, *CCF* congestive cardiac failure, *PHT* pulmonary hypertension, *ICU* intensive care unit, *SD* standard deviation, *DS* Down syndrome, *FSDs* feeding and swallowing difficulties

The medical profile (type of cardiac defect, disease severity, presence of complicated CHD, FSDs and a genetic disorder) and surgical outcomes (intensive care and HLOS, cardiopulmonary bypass time and occurrence of postoperative complications) were comparable for children with and without undernutrition. Undernourished children however tended to be more susceptible to postoperative infections. Nearly all undernourished children with FSDs (85.7%) had CHD complicated by CCF (Table 2).

**Fig. 2:**
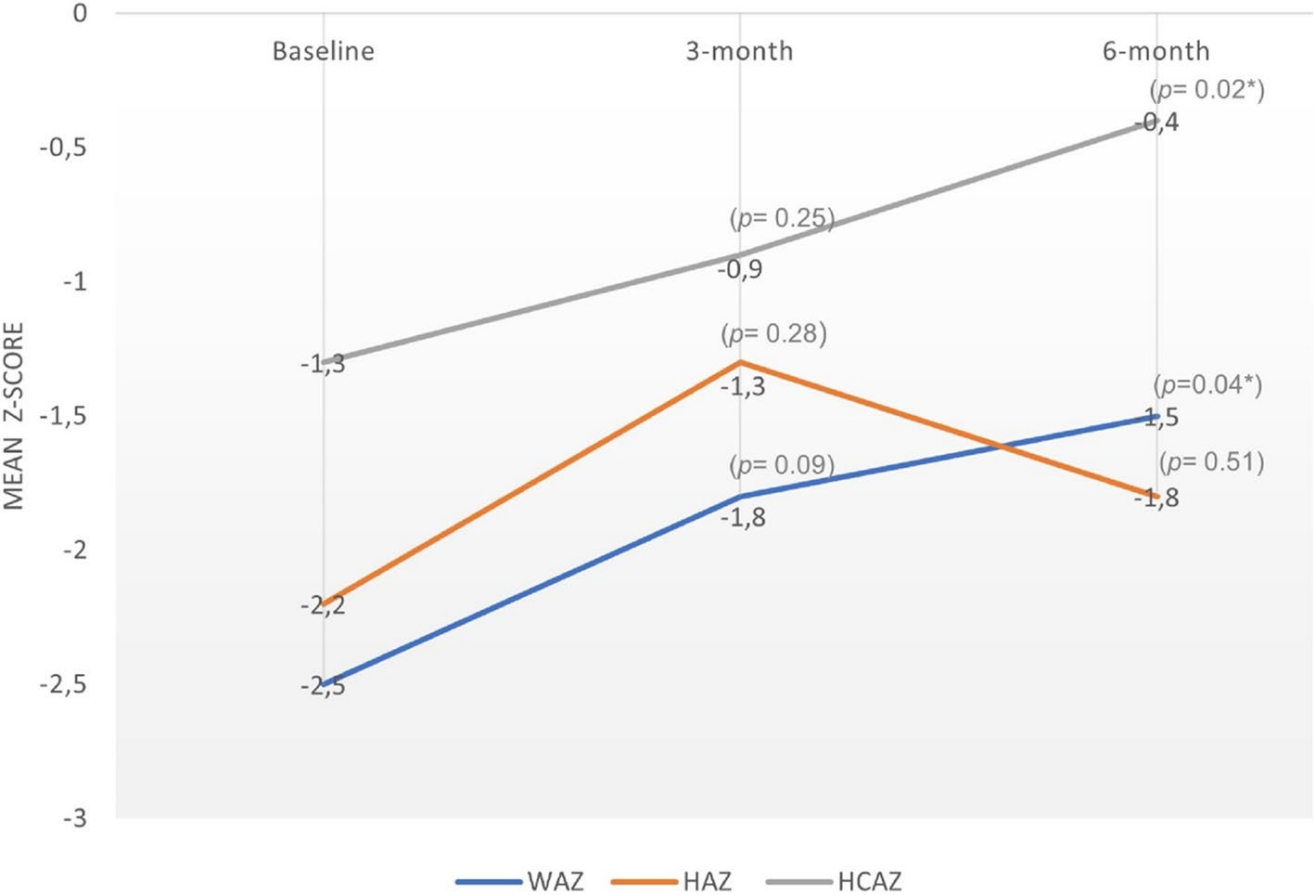
Mean z-scores for growth indices with changes in growth over time

Anthropometric data is presented as z-scores for weight-for-age, height-for-age, and head circumference-for-age at baseline (prior to cardiac surgery), and at 3-months and 6-month post-cardiac surgery (Fig. 2). In turn z-scores were used to identify the proportion of children with underweight, stunting and microcephaly at the various time points across the study (Table 3).

**Table 3:** Classification of growth outcomes across the study

Variable	Prior to surgery		Post-cardiac surgery			
	Baseline (***n*** = 40)		3- month (***n*** = 25)		6-month (***n*** = 22)	
	Frequency	%	Frequency	%	Frequency	%
Weight-for-age						
Severe underweight (Z score < − 3)	17	42.5	6	24	7	31.8
Moderate underweight (Z score < − 2)	10	25	8	32	2	9.1
Normal (Score > − 2 and < 2)	13	32.5	11	44	13	59.1
Height-for age						
Severe stunting (z-score < −3)	13	32.5	4	16	4	18.2
Moderate stunting (z-score < − 2)	5	12.5	5	20	4	18.2
Normal (z-score > −2 and < 2)	22	55	16	64	14	63.6
Microcephaly						
Microcephaly (z-score < −2)	10	25	5	20	3	13.6
Normal (z-score > −2 to < 2)	29	72.5	18	72	17	77.3
Macrocephaly (z-score > 2)	1	2.5	2	8	2	9.1
Summary of growth failures across the study						
Underweight (z-score < − 2)	27	68	14	56	9	40.9
Stunting (z-score < −2)	18	45	9	36	8	36.4
Microcephaly (z-score < −2)	10	25	5	20	3	13.6

Growth status improved across all growth indices from baseline to 6-months post-cardiac surgery, and by 3-months and 6-months post-cardiac surgery the mean z-scores for all growth indices fell within the acceptable range (z-score > −2 to 2). There was significant improvement in both weight-for-age (*p* = 0.04) and head circumference for-age (*p* = 0.02) from baseline to 6-months post-cardiac surgery

Despite considerable catch-up growth, complete catch-up growth had not yet occurred by 6 -months post-cardiac surgery for the sample as evidenced by the mean z-scores for all growth indices still falling below the 50th percentile (z-score of 0) (Fig. 2). By 6-months after cardiac surgery more than a third of the children were still undernourished, with 40.9% of the children being underweight (*n* = 9) and 36.4% stunted (*n* = 3). Most of the children who were underweight (*n* = 7) were severely underweight (Table 3).

Poor motor development prior to cardiac surgery was significantly associated with underweight (*p* = 0.04). Parent-reported feeding difficulties were not shown to be significantly associated with either poorer HRQOL or increased parenting stress in our study (Table 4).

**Table 4:** Association between undernutrition and feeding difficulties and outcomes of interest

Variables	*P*-values		
	Prior to cardiac surgery	Post-cardiac surgery	
	Baseline	3-month	6-month
Bayley-III cognitive scores			
Growth (weight-for-age)	0.32	0.49	0.26
Bayley-III language scores			
Growth (weight-for-age)	0.06	0.11	0.48
Bayley-III motor scores			
Growth (weight-for-age)	0. 04*	0.20	0.40
Health-related quality of life			
Growth (weight-for-age)	0.96	0.51	0.82
Parent reported feeding difficulties	0.13	0.59	0.58
Parenting stress			
Growth (weight-for-age)	0.89	0.28	0.62
Parent reported feeding difficulties	0.79	0.69	0.33

Indication of statistical significance: * *p* < 0.05

Growth outcomes for children with and without DS were comparable across all growth indices, including weight-for-age (*p* = 0.35), height-for-age (*p* = 0.64), and head circumference for age (*p* = 0.64), when DS-specific growth charts were used to determine the growth status of the children with DS (Table 5). Similarly, the growth outcomes of children with cyanotic and acyanotic defects were also found to be comparable across all growth indices, including weight-for-age (*p* = 1.00), height-for-age (*p* = 0.91), and head circumference-for-age (*p* = 0.33) (Table 6).

**Table 5:** Growth outcomes for children with CHD with and without Down syndrome

Variable	Before cardiac surgery			After cardiac surgery					
	Baseline			3-months			6-months		
	CHD with DS	CHD alone	***p***-value	CHD with DS	CHD alone	p-value	CHD with DS	CHD alone	***p***-value
Baseline	(n = 10)	(n = 30)		(***n*** = 5)	(***n*** = 20)		(n = 5)	(***n*** = 17)	
WAZ									
Mean and SD	−2.3 (±1.6)	−2.6 (±1.5)	0.59	−1.1 (±2.6)	− 2.0 (±1.4)	0.29	−0.9 (± 2.1)	− 1.8 (±1.8)	0.35
HAZ									
Mean and SD	−1.5 (±3.0)	−2.4 (±2.3)	0.32	−2.2 (±2.5)	−1.1 (±1.5)	0.21	−2.1 (±1.4)	−1.7 (±1.7)	0.64
HCAZ									
Mean and SD	−1.6 (±0.9)	−1.3 (±1.7)	0.60	−1.8 (±1.9)	−0.6 (±1.7)	0.18	−2.1 (±1.4)	−1.7 (±1.7)	0.64

*WAZ* weight-for- age z-score, *HAZ* height-for-age z-score and *HCAZ* gead circumference-for-age z-score, *CHD* congenital heart disease, *DS* Down syndrome

Indication of statistical significance: * *p* < 0.05

**Table 6:** Growth outcomes for children with cyanotic and acyanotic heart defects

Variable	Before cardiac surgery			After cardiac surgery					
	Baseline			3-months			6-months		
	Cyanotic	Acyanotic	***p***-value	Cyanotic	Acyanotic	p-value	Cyanotic	Acyanotic	***p***-value
Baseline	(***n*** = 8)	(***n*** = 32)		(***n*** = 4)	(***n*** = 21)		(***n*** = 4)	(***n*** = 18)	
WAZ									
Mean and SD	−2.5 (±1.4)	−2.5 (±1.5)	1.00	−3.0 (±0.8)	−1.6 (±1.7)	0.12	−1.8 (±2.5)	−1.8 (±1.6)	1.00
HAZ									
Mean and SD	−3.3 (±2.8)	−1.9 (±2.3)	0.15	−1.2 (±1.6)	− 1.6 (±1.9)	0.70	− 1.7 (±1.8)	−1.8 (±1.6)	0.91
HCAZ									
Mean and SD	0.2 (±1.7)	−1.1 (±1.7)	0.06	0.2 (±1.7)	−1.1 (±1.7)	0.17	0.5 (±1.3)	−0.5 (±1.9)	0.33

*WAZ* weight-for- age z-score, *HAZ* height-for-age z-score and *HCAZ* head circumference-for-age z-score

Indication of statistical significance: * *p* < 0.05

## Discussion

Adequate nutrition is essential for physical growth especially during the critical period of early childhood [35, 74]. Persistent malnutrition in early childhood is associated with poorer overall health, neurodevelopment, and lower educational and economic attainment across the life course [53, 75]. Consequentially the 2030 sustainable development agenda targets ending hunger and improving nutrition for all children, including children with CHD, in order to improve early childhood development outcomes [76, 77].

Most of the children in our study were underweight (68%) and a high number stunted (45%) before their cardiac surgery. In fact, the prevalence of malnutrition (undernutrition) was far higher than the prevalence of underweight and stunting reported for South African children under the age of 5 years at 5.5 and 23% respectively [78, 79]. By implication children with CHD in central SA were nearly 12 times more likely to be underweight and twice as likely to be stunted compared to their healthy same-aged peers.

On average six children 30 months and younger underwent cardiac surgery monthly at our cardiac center. Over the study 19 children (47.5%) were lost to follow-up for reasons including post-operative mortality (*n* = 6), non-compliance with follow-up (*n* = 9) and family relocation (*n* = 1). Measures taken to limit loss to follow-up, inconvenience and the financial burden on families included scheduling follow-up visits to coincide with routine cardiology clinic visits, mothers were sent Short Message Service (SMS) text messages and contacted telephonically to remind them of follow-up appointments, and a financial contribution was made towards covering study-related travel costs. All children included in the study accessed public healthcare. Public healthcare for South African children under the age of 6 years is free, with the cost of cardiac care being covered by the government [80]. Despite cardiac care and inter-hospital transport being provided by the government, families from low-income environments experience increased financial strain resulting from out-of-pocket expenses related to their child’s ongoing cardiac care (accommodation, food, transport, and phone charges) and/ or a loss of income (having to stay home to care for the child or having to miss work to meet follow-up care appointments). In many cases the added financial strain contributes to families being non-compliant with follow-up cardiac care [40, 81].

The attrition rate following cardiac surgery though high, was comparable with that reported in HICs and LMICs [81–85]. Factors contributing to non-compliance with follow-up in our study included the distance to be travelled to access cardiac services, unreliable inter-hospital transport services, cost and the time required of families to attend follow-up visits and a lack of understanding on the part of families of the importance of regular cardiac follow-up. It is important to mention the high loss to follow-up and its contributing factors as these would be important considerations when designing, implementing, and monitoring nutritional support and feeding therapy interventions.

On comparing our findings on malnutrition (undernutrition) with the globally reported prevalence for underweight (27.4%) and stunting (24.4%) in children with CHD, children with CHD in central South Africa are two-and-a half times more likely to be underweight and twice as likely to be stunted [17]. This keeping in mind that the global prevalence of underweight and stunting in children with CHD is already significantly higher than estimates provided by the World Health Organization (WHO)/World Bank Joint Child Malnutrition Estimates group in 2020, where 12.6% of children globally were reported to be underweight and 22.0% stunted [28, 29].

Despite head circumference being well-established as a strong indicator for both brain development and nutritional status, published literature has rarely reported on head circumference-for-age z-scores (HCAZ) as an index for growth status in children with CHD [86, 87]. The preoperative prevalence of microcephaly (25%) in children with CHD in the current study falls on the higher end of a widely variable reported prevalence of 3 to 36% [8, 88, 89]. The high prevalence of microcephaly prior to cardiac surgery may be explained by the greater extent of the malnutrition (undernutrition) of children in our study due to low-income environments [90, 91], in combination with low birth weight (LBW) which is likely to have been caused by intrauterine growth restriction (IUGR) caused in turn by impaired cerebral blood flow [92, 93]. Poor maternal nutrition, although not measured in this study, may also have contributed to LBW [94, 95]. Furthermore, genetic comorbidity in the form of DS may also have been a contributory factor [96].

The high prevalence of both underweight and stunting in children with CHD in central SA is consistent with the prevalence reported in other studies in Africa [14, 34, 36, 97, 98] and other LMICs [31, 38, 39], affirming that the presence of CHD not only increases the likelihood of malnutrition (undernutrition), but also increases its severity in children living in low-income environments [11, 31, 34]. Moreover, this is corroborated by a recent systematic review and meta-analysis that concluded that there are significant differences in the prevalence of underweight and stunting in children with CHD across continents, with Africa having the highest rates of underweight and stunting [17].

The high prevalence of malnutrition in our study can be explained by several factors including hemodynamic factors related to the cardiac defect, the presence of CCF (21.4%), reversible PHT (60.7%), recurrent respiratory infections requiring hospitalization prior to surgery (62.5%), inadequate nutritional intake due to FSDs (25%), and LBW (25%). The contributing factors are consistent with those reported in previous studies from LMICs [14, 30, 32, 33, 35, 37, 39, 98, 99]. Older age at first cardiac surgery in the current study (median age of 7.4 months) may also have pronounced the extent of the malnutrition (undernutrition) preoperatively [30, 31, 97].

A quarter of the children in our study had reported feeding difficulties. Poor feeding ability is common in children with CHD and is known to contribute to growth failure [6, 7, 13, 16]. Most of the undernourished children with reported FSDs had CHD complicated by CCF. Children with CHD associated with CCF are likely to have a poor appetite, take longer to feed due to shortness of breath, and an enlarged heart may result in less room for the stomach to expand to hold food [100, 101]. Furthermore, it is also known that children with DS have feeding difficulties due to hypotonia and a protuberant tongue which may have contributed to feeding difficulties and reduced caloric intake in this sub-group of children [7, 102].

The medical profile and surgical outcomes for children with and without malnutrition were comparable. Undernourished children however appeared to be more susceptible to postoperative infections. Undernutrition makes a child more vulnerable to infection by reducing the gut barrier and immune function, altering the microbiome, and causing chronic inflammation. The risk of infection is directly related to the severity of the undernutrition [103, 104].

It is likely that socioeconomic factors contributed to the prevalence and extent of the growth failure in our study sample [17, 30]. Most families came from low-income environments, had low levels of education (average of grade 9-11), with a high rate of unemployment (33%) amongst fathers. The high rate of unemployment is reflective of the poor economic climate in SA where unemployment rates are similarly high at around 35% [105]. When employed, fathers tended to be the primary breadwinner. Consistent with previous studies, most mothers did not work outside of the home in order to take care of domestic responsibilities, and to care for the child with CHD and other siblings [106, 107]. Malnutrition and low-income environments are closely linked as people living in poverty often face considerable financial constraints, which prevent them from being able to access safe, sufficient, and nutritious food [34, 58]. It is likely that findings would be similar for children with CHD living in low-income environments in HICs.

Consistent with previous studies, this study saw initial reduced weight attainment reflected by the lower weight-for-age z-scores (WAZ) prior to cardiac surgery, as seen in acute malnutrition. Some children demonstrated more sustained inadequate nutrition resulting in stunting which was reflected by the lower height-for-age z-scores (HAZ) [7]. The median age of children at their first cardiac surgery of 7.5 months (with an upper age range of 20.9 months) may also in part explain the high prevalence of stunting prior to cardiac surgery. In addition, abnormal fetal blood flow and likely poor maternal nutrition may have contributed to IUGR and LBW which in turn may also have contributed to the chronic malnutrition seen prior to cardiac surgery [12, 108, 109].

Hypotonia was prevalent in children prior to cardiac surgery, and present in all children with DS. Hypotonia resolved in children without DS by the 6-months after cardiac surgery. Hypotonia is typically present in children with DS [110, 111]. Hypotonia in children without DS is likely due to undernutrition and chronic disease which alters the body composition resulting in a loss of lean muscle mass. A loss of lean muscle mass is associated with muscle weakness and low muscle tone [112].

### Growth catch-up following cardiac surgery

Growth indices for weight, height and head circumference all improved by 6-months post-cardiac surgery, with significant improvements in both WAZ (*p* = 0.04) and HCAZ (*p* = 0.02) by 6-months post-cardiac surgery. The initial improvement in the mean HAZ at 3-months post-cardiac surgery followed by a slight a decline by 6-months is likely attributable to the small sample size. HAZ however still improved from baseline to 6-months post-cardiac surgery, though the improvement was not statistically significant. By 3-months and 6-months post-cardiac surgery, the mean z-scores for WAZ, HAZ and HCAZ fell within the acceptable range. However, complete catch-up growth to had not yet occurred, as the mean z-scores were still below the 50th percentile (z-score of zero). The considerable short term catch-up growth in the current study is likely owing to the correction of the hemodynamic alterations caused by the CHD [11]. Our finding is consistent with several studies that reported the prevalence of post-operative malnutrition decreased significantly over time (often referred to as catch-up growth), with significant improvements for height and weight, taking place within 3-months to 12-months after cardiac surgery [7, 8, 11, 12, 17, 30, 38].

Despite improved growth post-cardiac surgery several children still presented with ongoing growth failure in the form of underweight (*n* = 9, 40.9%) and stunting (*n* = 8; 36.4%) at 6-months post-cardiac surgery. There are limited studies reporting on postoperative growth outcomes in children with CHD; however, the prevalence of ongoing growth failure in our study fell within the reported prevalence for post-operative growth failure of 15.9 to 59% [12, 14, 27, 57, 81, 83]. The prevalence of persistent growth failure in our study can be considered high especially as most children underwent definitive corrective cardiac surgeries. This would suggest that factors other than the CHD itself contributed to growth failure. Patient-related factors including poor preoperative growth, feeding difficulties, presence of genetic comorbidity and socioeconomic factors such as poor living environments and possible food insecurity likely contributed to the persistent growth failure [7, 18]. It has also been reported that in cases where the heart defect is repaired beyond infancy, more limited catch-up growth can take place [17].

### Association of growth failure and poorer gross motor developmental performance

Growth failure before cardiac surgery was significantly associated (*p* = 0.04) with poorer motor performance. Available evidence confirms that undernutrition can compromise all areas of a child’s development both in the short and longer term [113]. Studies investigating the relationship between undernutrition and neurodevelopmental outcome are however lacking [114]. Moreover, growth failure is a recognized risk for poorer neurodevelopmental outcome in children with CHD [115]. Consistent with published findings, children in our study who were undernourished compensated for their lack of dietary energy and lean body mass by decreasing their energy expenditure through reduced physical activity [116]. Undernourished children demonstrated poorer physical endurance and fatigued more easily when engaged in age appropriate activities [117, 118]. Decreased physical activity and hypotonia preoperatively negatively impacted the acquisition of motor skills and decreased environmental exploration, which in turn may also have negatively affected the acquisition of cognitive skills [116, 119, 120]. Contrastingly, growth was not found to be strongly associated with developmental performance at either 3-months or 6-months post-cardiac surgery. This is likely explained by the significantly improved nutritional status of the children by 3-months postoperatively, as well as the resolution of hypotonia in almost all children without DS, improved cardiovascular endurance, and less maternal over-protection [83, 121].

### Growth outcomes in special populations

Growth outcomes for children with cyanotic and acyanotic defects in our study were comparable. Likewise, several studies report insignificant differences in the growth outcomes for children with cyanotic and acyanotic defects [17, 18, 34, 98]. Nevertheless there are also several studies that have reported poorer growth outcomes for children with cyanotic defects [7, 14, 37, 97]. A reason for the contradictory findings across studies could be the varied representation of cyanotic defects in the individual study samples. In our study none of the children had single ventricle physiologies and children with cyanotic defects constituted only a small portion of the sample, which may have contributed to our finding of comparable growth outcomes.

Growth outcomes for children without and with DS were found to be similar when DS-specific growth standards were used for children with DS, allowing for fairer comparison with their peers. Comparing our findings with other published data proved challenging as previous studies used standard growth charts to determine growth outcomes for children with CHD with DS [12, 122]. It is possible that these studies may have overestimated the extent of the growth failure for children with CHD with DS, contributing to their finding of significant differences in growth between children with CHD with and without DS. A more recent study, similarly to our study, reported that although infants with genetic disorders weighed less, were shorter and had a smaller head circumference the differences were statistically insignificant when compared with child with CHD without genetic disorders [60].

### Study limitations

The findings in this study need to be interpreted in the light of several limitations. This study only reports on a single center’s experience, making it difficult to generalize the finding to the larger population of children with CHD in SA and beyond. It is acknowledged that the sample size in the current study was small, however almost all children from this single cardiac center, who met the inclusion criteria, were recruited over the study period. The small sub-group sizes make it difficult to draw definitive conclusions from the formal statistical analyses of between group differences in growth outcomes for children with cyanotic and acyanotic defects, and those with and without DS. Despite this limitation, it was still considered important to report on trends or tendencies in outcomes between these sub-groups of interest. In retrospect, considering that catch-up growth is reported to take place until 12-months after cardiac surgery, the follow-up period of 6-months post cardiac surgery is likely to have been too short to establish if complete catch-up growth took place [7, 8, 11, 12, 17, 30, 38].

This study only included a self-report from mothers on the presence of feeding difficulties. This may have resulted in some children with feeding and swallowing problems going unidentified. In retrospect a supplemental feeding assessment by a trained speech therapist may have been beneficial in definitively identifying those children with feeding dysfunction that likely negatively affected their growth [13].

Despite the recognized limitations the current study still provides valuable first data on growth outcomes of children with CHD in central SA from before cardiac surgery and in the short term post-operatively. This study may also provide some insights on malnutrition (undernutrition) for clinicians working with children with CHD living in low-income environments in HICs.

### Clinical practice and future research recommendations

The high prevalence of malnutrition (undernutrition) and feeding difficulties in children with CHD in central SA prior to cardiac surgery emphasizes the importance of ensuring adequate and routine preoperative assessment of growth and feeding ability by a dietician and speech therapist who are to be considered integral members of an interdisciplinary cardiac care team. Where malnutrition and feeding difficulties present, interventions including nutritional education and support and feeding therapy should be provided to improve nutritional status and post-operative outcomes [11, 13, 18, 31]. This study also provided evidence in support of regular monitoring of catch-up growth after cardiac surgery for a period of at least 12 months to identify those children who persist with poor growth. Where catch up growth does not occur, possible modifiable causes need to be identified and addressed [6, 11].

Recommendations for future research include the evaluation of growth status in a larger sample including adequate representation of special populations such as children with cyanotic lesions and those with genetic comorbidity. Children with genetic comorbidity also need to be included in research investigating growth outcomes for children with CHD to better understand the growth outcomes for this sub-group of children.

## Conclusions

Most children with CHD in central South Africa are malnourished (undernourished) prior to cardiac surgery, which in turn negatively impacted their motor development. Despite significant catch-up growth occurring postoperatively, complete catch-up growth had not yet taken place by 6-months post-cardiac surgery. A diagnosis of CHD therefore warrants regular monitoring of all growth indices by the cardiac care team to identify those children at risk for or presenting with growth failure, facilitating referral to a dietician for nutritional education and support. Likewise feeding skills should also be assessed by a speech therapist where feeding difficulties are suspected to identify those children likely to benefit from feeding therapy to optimize nutritional status and postoperative outcomes. Furthermore, regular monitoring of catch-up growth after cardiac surgery for a period of at least 12 months is recommended to identify children who persist with poor growth.

## Data Availability

The dataset used and/or analyzed during the current study are available from the corresponding author on reasonable request.

## Abbreviations

ANOVA: Analysis of variance
AS: Aortic stenosis
AVSD: Atrioventricular septal defect
Bayley-III: Bayley Scales of Infant and Toddler Development, third edition
CCF: Congestive cardiac failure
CHD: Congenital heart disease
CLP: Cleft lip palate
COA: Coarctation of the aorta
DS: Down Syndrome
DORV: Double outlet right ventricle
FSDs: Feeding and swallowing difficulties
GERD: Gastroesophageal Reflux Disease
HAZ: Height-for-age z-score
HCAZ: Head circumference-for-age z-score
HIC: High income country
HLHS: Hyperplastic left heart syndrome
HLOS: Hospital length of stay
HRQOL: Health-related quality of life
ICU: Intensive care unit
IUGR: Intrauterine growth restriction
LMIC: Low-to-middle income country
LBW: Low birth weight
PDA: Patent ductus arteriosus
PHT: Pulmonary hypertension
SA: South Africa
SD: Standard deviation
SMS: Short message service
TA: Tricuspid atresia
TAPVD: Total anomalous pulmonary venous drainage
TGA: Transposition of the great arteries
TOF: Tetralogy of Fallot
UK: United Kingdom
UNICEF: United Nations Children’s Fund
VSD: Ventricular septal defect
US: United States
WAZ: Weight-for-age z score
WHO: World Health Organization
