# Correction: Undernutrition in young children with congenital heart disease undergoing cardiac surgery in a low‑income environment

**DOI:** 10.1186/s12887-024-04613-5

**Published:** 2024-02-12

**Authors:** Robyn Smith, Veronica Ntsiea, Stephen Brown, Joanne Potterton

**Affiliations:** 1 Department of Physiotherapy, Faculty of Health Sciences, University of the Witwatersrand, Johannesburg, South Africa; 2 Department of Pediatrics and Child Health, University of the Free State, Bloemfontein, South Africa; 3 School of Health and Rehabilitation Sciences, University of the Free State, Bloemfontein, South Africa

**Correction: *BMC Pediatr*** **24, 73 (2024)**

10.1186/s12887-023-04508-x

Following publication of the original article [1], the authors reported that their names in the author list, which are presented as “Smith Robyn, Ntsiea Veronica, Brown Stephen and Potterton Joanne“, are incorrect and should be corrected to “Robyn Smith, Veronica Ntsiea, Stephen Brown and Joanne Potterton“.
